# Zanubrutinib in Japanese treatment-naive and relapsed/refractory patients with Waldenström macroglobulinemia and CLL/SLL

**DOI:** 10.1007/s12185-025-03925-1

**Published:** 2025-02-17

**Authors:** Koji Izutsu, Takayuki Ishikawa, Kazuyuki Shimada, Kohmei Kubo, Takeshi Kondo, Katsuya Fujimoto, Tomoaki Fujisaki, Shingo Kurahashi, Koji Nagafuji, Rika Sakai, Tatsuro Jo, Tomonori Nakazato, Kazutaka Sunami, Senji Kasahara, Aileen Cohen, Motohisa Takai, Jinhua Zhong, Masahiro Takeuchi

**Affiliations:** 1 National Cancer Center Hospital, 5-1-1 Tsukiji, Chuo-ku, Tokyo Japan; 2 Kobe City Medical Center General Hospital, Kobe, Japan; 3 Nagoya University Hospital, Nagoya, Japan; 4 Aomori Prefectural Central Hospital, Aomori, Japan; 5 Aiiku Hospital, Sapporo, Japan; 6 NHO Hokkaido Cancer Center, Sapporo, Japan; 7 Matsuyama Red Cross Hospital, Matsuyama, Japan; 8 Toyohashi Municipal Hospital, Toyohashi, Japan; 9 Kurume University Hospital, Kurume, Japan; 10 Kanagawa Cancer Center, Yokohama, Japan; 11 Japanese Red Cross Nagasaki Genbaku Hospital, Nagasaki, Japan; 12 Yokohama Municipal Citizen’s Hospital, Yokohama, Japan; 13 National Hospital Organization Okayama Medical Center, Okayama, Japan; 14 Gifu Municipal Hospital, Gifu, Japan; 15 BeiGene USA, Inc., San Mateo, CA USA; 16 BeiGene (Shanghai) Co., Ltd., Shanghai, China; 17 Chiba-Ken Cancer Center, Chiba, Japan

**Keywords:** Waldenström Macroglobulinemia, Chronic Lymphocytic Leukemia/Small Lymphocytic Lymphoma, Zanubrutinib, Bruton tyrosine kinase, Japanese

## Abstract

Zanubrutinib is a selective second-generation Bruton tyrosine kinase inhibitor approved in various B-cell malignancies globally. The phase 1/2 BGB-3111-111 study evaluated the efficacy and safety of zanubrutinib 160 mg twice daily orally in Japanese patients with treatment-naive or relapsed/refractory mature B-cell malignancies. Here, efficacy results from Part 2 in chronic lymphocytic leukemia/small lymphocytic lymphoma (CLL/SLL; n = 19) and Waldenström macroglobulinemia (WM; n = 19), and safety results from Parts 1 (N = 6) and 2 (N = 49) are presented, with the first dose between 30 January, 2020, and 31 October, 2022. As of 10 May, 2023, investigator-assessed overall response rates were 100% (19/19) and 94.7% (18/19) in CLL/SLL and WM, respectively, with median follow-up of 27.9 and 26.8 months; 24-month progression-free survival rates were 71.4% and 100% in treatment-naive and relapsed/refractory CLL/SLL and 83.9% and 100% in treatment-naive and relapsed/refractory WM, respectively. In patients with B-cell malignancies, any-grade treatment-emergent adverse events (TEAEs) occurred in 53 (96.4%) and serious TEAEs in 18 (32.7%). Common TEAEs were platelet count decreased (18.2%), pyrexia (18.2%), COVID-19 (14.5%), and neutrophil count decreased (12.7%). With median follow-up > 2 years, zanubrutinib demonstrated durable efficacy in Japanese patients with CLL/SLL or WM and a favorable safety profile consistent with global phase 3 studies.

**Supplementary Information:**

The online version contains supplementary material available at 10.1007/s12185-025-03925-1.

## Introduction

Chronic lymphocytic leukemia/small lymphocytic lymphoma (CLL/SLL) and Waldenström macroglobulinemia (WM) are B-cell malignancies that cannot be cured with conventional cytotoxic chemotherapy [1–3]. The treatment landscape for CLL/SLL and WM has evolved over the past decade. One of the latest evolutions in treatment paradigms is the introduction of Bruton tyrosine kinase (BTK) inhibitors, which inhibit the BTK enzyme, a key intracellular mediator of B-cell receptor signaling that promotes growth and survival of malignant B cells [4–6]. The first-generation BTK inhibitor, ibrutinib, is an orally administered medication approved in Japan for the treatment of both CLL and WM; however, it may be associated with treatment resistance and an increased frequency of cardiovascular adverse events (AEs), including atrial fibrillation and hypertension [7]. Although BTK inhibitors have improved treatment outcomes in patients with CLL and WM, there is still an unmet need for safer and more effective treatment options.

Zanubrutinib is a potent, selective, irreversible, second-generation BTK inhibitor designed to maximize BTK occupancy and minimize off-target kinase inhibition [8]. Zanubrutinib has demonstrated superior safety and efficacy versus bendamustine plus rituximab in patients with treatment-naive (TN) CLL/SLL [9], versus ibrutinib in patients with relapsed/refractory (R/R) CLL/SLL [10], and showed comparable efficacy with an improved safety profile versus ibrutinib in patients with WM [11] in global phase 3 studies. Zanubrutinib is approved for these indications in the US [12], Europe [13], China [14], and more than 70 countries overall.

The BGB-3111-111 study (NCT04172246) is an ongoing, multicenter, open-label, phase 1/2 study designed to assess the safety and efficacy of zanubrutinib in Japanese patients with B-cell malignancies. Here, the efficacy and safety findings from this study in Japanese patients with TN or R/R CLL/SLL or WM are reported.

## Materials and methods

### Study design and patients

The study consisted of 2 parts, and the trial design is shown in Supplementary Fig. 1. Patients eligible to participate in the study were Japanese adults (age ≥ 20 years) with adequate organ function; an Eastern Cooperative Oncology Group performance status (ECOG PS) of 0–2; and a confirmed diagnosis of CLL/SLL, mantle cell lymphoma (MCL), WM, marginal zone lymphoma, or follicular lymphoma in Part 1 and TN or R/R CLL/SLL, TN or R/R WM, or R/R MCL in Part 2.

Patients were ineligible if they had prior allogeneic stem cell transplant or prior therapy with B-cell receptor inhibitors (eg, BTK inhibitors, phosphoinositide 3-kinase delta inhibitors, and/or spleen tyrosine kinase inhibitors) or BCL2 inhibitors. Additional inclusion and exclusion criteria are available in the Supplementary Methods: Selection of Study Population.

Baseline assessments were conducted at screening, which included assessment of the mutation status of *MYD88/CXCR4* in patients with WM, and del(17p) and immunoglobulin heavy chain variable region (IGHV) in patients with CLL/SLL. IGHV mutation status was evaluated by next-generation sequencing, which detects the clonal population of B-lymphocytes in blood samples collected from patients for analysis of the VDJ segment of the immunoglobulin heavy chain (IgH) gene. The mutation status of *MYD88* and *CXCR4* were also evaluated by next-generation sequencing using non-enriched bone marrow aspirate samples.

Part 1 assessed the safety, tolerability, and pharmacokinetics of zanubrutinib in patients with R/R mature B-cell malignancies. Six patients received a single dose of zanubrutinib 160 mg orally, followed by a 24-h washout period. After the washout, these patients received zanubrutinib 160 mg orally twice daily. After the first 6 patients had been treated for 28 days, a safety monitoring committee reviewed the safety and tolerability data from these patients. The committee confirmed the safety and tolerability of zanubrutinib after evaluating dose-limiting toxicity (DLT) events (Supplementary Methods: Dose-Limiting Toxicity) in patients enrolled in Part 1, and enrollment for Part 2 was initiated.

Part 2 assessed the efficacy, safety, tolerability, and pharmacokinetics of zanubrutinib in four disease-specific cohorts: R/R MCL, TN CLL/SLL, R/R CLL/SLL, and TN or R/R WM. Patients received zanubrutinib 160 mg orally twice daily continuously until disease progression, unacceptable toxicity, death, withdrawal of consent, loss to follow-up, end of study, investigator’s decision, or study termination by sponsor. The planned enrollment was 36–53 patients for Part 2.

### Objectives and endpoints

The objectives of Part 1 were to evaluate the safety and tolerability of oral zanubrutinib 160 mg twice daily and the pharmacokinetic profile of a single dose and multiple doses of zanubrutinib 160 mg. Patients in Part 1 were evaluated for DLTs, treatment-emergent AEs (TEAEs), and pharmacokinetics.

The objective of Part 2 was to assess the efficacy and evaluate the safety and tolerability of zanubrutinib 160 mg twice daily. The primary endpoint was overall response rate (ORR) assessed by independent review committee (IRC), defined as the proportion of patients achieving the following: for CLL, complete response (CR), CR with incomplete bone marrow recovery, partial response (PR), or PR with lymphocytosis (PR-L) per the 2018 International Workshop on Chronic Lymphocytosis Leukemia guidelines [15, 16]; for SLL, PR or better per the Lugano classification for non-Hodgkin lymphoma [17]; and for WM, CR, very good PR (VGPR), PR, or minor response per the response criteria updated at the sixth International Workshop on WM [18].

Secondary endpoints included progression-free survival (PFS), duration of response (DoR), time to response by IRC, ORR assessed by investigator, overall survival (OS), and safety (TEAEs). TEAEs were assessed and graded based on the National Cancer Institute Common Terminology Criteria for Adverse Events v5.0, except for hematologic toxicities in patients with CLL/SLL, for which the grading scale from the International Workshop on CLL was used. Details of the AE evaluation are available in the Supplementary Methods: Adverse Events.

The investigator-assessed endpoints for CLL/SLL and WM in Part 2 were analyzed at the 10 May 2023 data cutoff and are presented; the MCL cohort was not analyzed for efficacy at this data cutoff. The safety analyses included all patients who received ≥ 1 dose of zanubrutinib, including patients with MCL and patients in Part 1.

### Statistical analysis

All patients who received at least 1 dose of zanubrutinib were included in the exposed analysis set and the safety and efficacy analyses. Demographic and baseline characteristics were summarized using descriptive statistics. All efficacy and safety analyses were descriptive, except the formal hierarchical fixed-sequence hypothesis testing performed in the WM cohort with an earlier data cutoff date of 10 May 2022. ORR in patients with WM was compared against a historical control rate [19] of 52% at a 1-sided alpha of 0.05, as described in the Supplementary Methods: Statistical Analysis. All statistical analysis was conducted using SAS Version 9.4 (SAS Institute, Inc., North Carolina, USA).

### Ethics and data sharing

This study was conducted in accordance with the Declaration of Helsinki and the International Conference on Harmonization Guidelines for Good Clinical Practice; written informed consent was obtained from each patient, and institutional review board approval was obtained at each study site.

BeiGene voluntarily shares anonymous data on completed studies responsibly and provides qualified scientific and medical researchers access to anonymous data and supporting clinical trial documentation for clinical trials in dossiers for medicines and indications after submission and approval in the US, China, and Europe. Clinical trials supporting subsequent local approvals, new indications, or combination products are eligible for sharing once corresponding regulatory approvals are achieved. BeiGene shares data only when permitted by applicable data privacy and security laws and regulations. In addition, data can only be shared when it is feasible to do so without compromising the privacy of study participants. Qualified researchers may submit data requests/research proposals for BeiGene review and consideration through BeiGene’s Clinical Trial Webpage at https://www.beigene.com/our-science-and-medicines/our-clinical-trials/.

## Results

### Patients

**Fig. 1:**
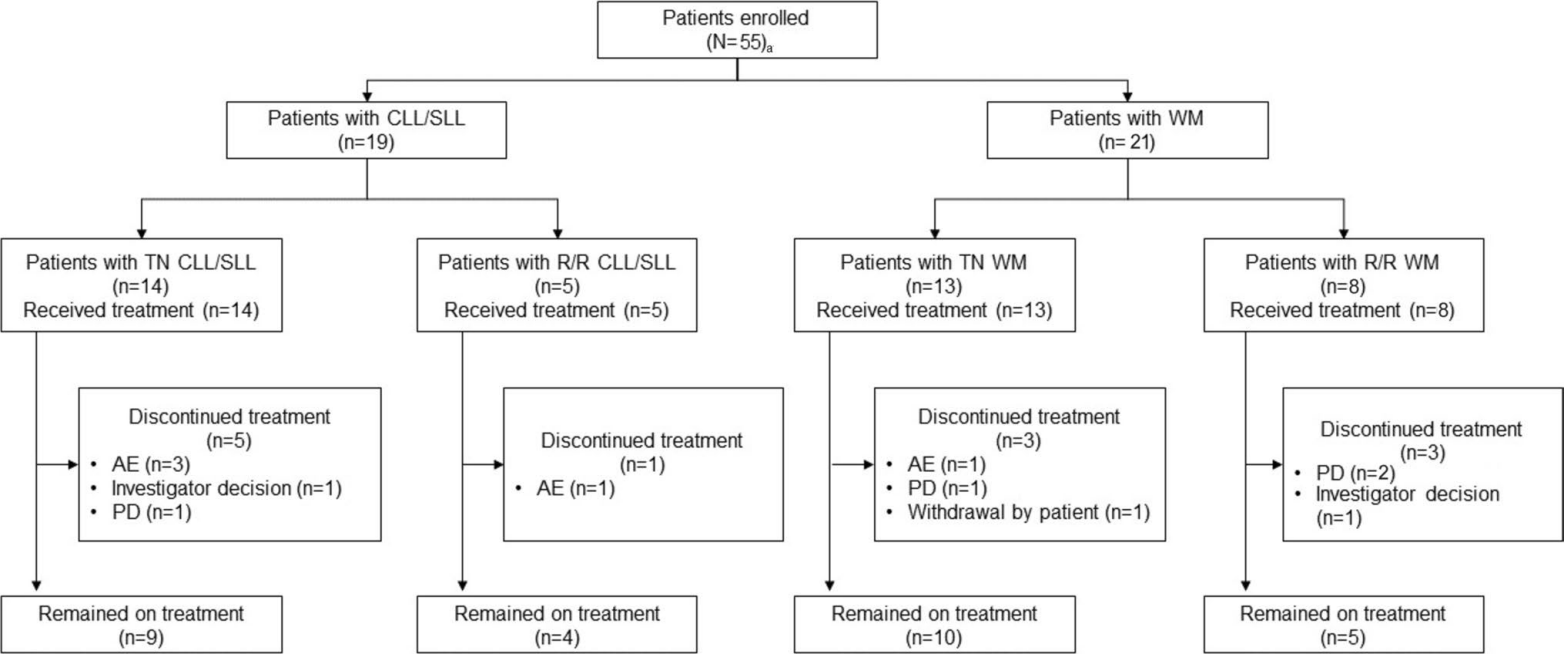
Patient Disposition. Data cutoff: 10 May 2023. ^a^ Includes patients from Part 1 (FL, n = 2; MZL, n = 1; MCL, n = 1) and the Part 2 R/R MCL cohort (n = 11). *AE* adverse event; *CLL/SLL* chronic lymphocytic leukemia/small lymphocytic lymphoma; *FL* follicular lymphoma; *MCL* mantle cell lymphoma; *MZL* marginal zone lymphoma; *PD* progressive disease; *R/R* relapsed/refractory; *TN* treatment naive; *WM* Waldenström macroglobulinemia

A total of 55 patients (6 in Part 1 and 49 in Part 2) were enrolled at the 10 May 2023 data cutoff, including 19 patients with CLL/SLL (TN, n = 14; R/R, n = 5) and 21 patients with WM (TN, n = 13; R/R, n = 8), with the first dose of zanubrutinib between 30 January 2020 and 31 October 2022 (Fig. 1). Most patients in both the CLL/SLL and WM subgroups were male and aged ≥ 65 years (Table 1). The median age was 71.0 years (range, 38–77 years) in patients with CLL/SLL and 69.0 years (range, 37–83 years) in those with WM. The median number of prior lines of therapy was 2 (range, 1–4) and 3.5 (range, 1–8) in patients with R/R CLL/SLL and R/R WM, respectively. Among patients with WM, 17 (81.0%) had an *MYD88*^L265P^ mutation and 4 (19.0%) had *CXCR4*^WHIM^ per next-generation sequencing; among patients with CLL/SLL, none had del(17p), as assessed by fluorescent in situ hybridization, and 12 patients (63.2%) had mutated IGHV.

**Table 1:** Demographics and Baseline Characteristics^a^

	CLL/SLL			WM			Total (N = 55)^b^
	TN (n = 14)	R/R (n = 5)	All (n = 19)	TN (n = 13)	R/R (n = 8)^c^	All (n = 21)^c^	
Age, median (range), years	67.5 (38–77)	72.0 (52–77)	71.0 (38–77)	71.0 (37–83)	67.5 (61–78)	69.0 (37–83)	71.0 (37–84)
≥ 65 years, n (%)	8 (57.1)	4 (80.0)	12 (63.2)	10 (76.9)	6 (75.0)	16 (76.2)	41 (74.5)
Sex, n (%)							
Male	10 (71.4)	4 (80.0)	14 (73.7)	6 (46.2)	5 (62.5)	11 (52.4)	38 (69.1)
Female	4 (28.6)	1 (20.0)	5 (26.3)	7 (53.8)	3 (37.5)	10 (47.6)	17 (30.9)
ECOG PS, n (%)							
0	12 (85.7)	5 (100)	17 (89.5)	10 (76.9)	5 (62.5)	15 (71.4)	44 (80.0)
1	2 (14.3)	0	2 (10.5)	3 (23.1)	3 (37.5)	6 (28.6)	11 (20.0)
No. of prior lines of therapy in patients with R/R disease, median (range)	–	2.0 (1–4)	–	–	3.5 (1–8)	–	2.0 (1–8)
Mutated* MYD88*^L265P^, n (%)^d^	–	–	–	11 (84.6)	6 (75.0)	17 (81.0)	–
*CXCR4*^WHIM^, n (%)^d^	–	–	–	4 (30.8)	0	4 (19.0)	–
Del(17p), n (%)	0	0	0	–	–	–	–
IGHV mutational status, n (%)							
Mutated	9 (64.3)	3 (60.0)	12 (63.2)	–	–	–	–
Unmutated	5 (35.7)	2 (40.0)	7 (36.8)	–	–	–	–

*CLL/SLL* chronic lymphocytic leukemia/small lymphocytic lymphoma; *ECOG PS* Eastern Cooperative Oncology Group performance status; *FL* follicular lymphoma; *MCL* mantle cell lymphoma; *MZL* marginal zone lymphoma; *NGS* next-generation sequencing; *R/R* relapsed/refractory; *TN* treatment naive; *WHIM* warts, hypogammaglobulinemia, immunodeficiency, and myelokathexis syndrome; *WM* Waldenström macroglobulinemia

^a^Data cutoff: 10 May 2023

^b^Includes patients from Part 1 (FL, n = 2; MZL, n = 1; MCL, n = 1) and the Part 2 R/R MCL cohort (n = 11)

^c^Includes 2 patients with WM from Part 1

^d^Genotype subgroups (*MYD88* per NGS; *CXCR4* per NGS) were determined using bone marrow samples collected

### Overall response

**Fig. 2:**
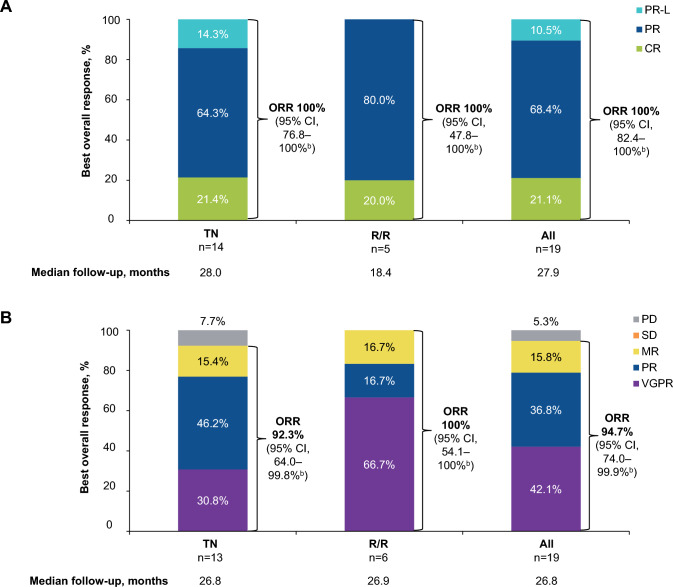
Best Overall Response Assessed by Investigator in Part 2 in Patients With **A** CLL/SLL and **B** WM^a^. ^a^Data cutoff: 10 May 2023. ^b^Estimated using Clopper-Pearson method. CLL/SLL chronic lymphocytic leukemia/small lymphocytic lymphoma; *CR* complete response; *MR* minor response; *ORR* overall response rate; *PD* progressive disease; *PR* partial response; *PR-L* partial response with lymphocytosis; *R/R* relapsed/refractory; *SD* stable disease; *TN* treatment naive; *VGPR* very good partial response; *WM* Waldenström macroglobulinemia

At the latest data cutoff of 10 May 2023, investigator-assessed ORR (PR-L or better) in CLL/SLL was 100% in both the TN and R/R groups and the overall CLL/SLL group, with a median follow-up of 28.0 and 18.4 months in the TN and R/R CLL/SLL groups, respectively (Fig. 2A). In the WM cohort, the investigator-assessed ORR (minor response or better) was 92.3% in the TN WM group, 100% in the R/R WM group, and 94.7% in the overall WM group, with a median follow-up of 26.8 and 26.9 months in the TN and R/R WM groups, respectively. The major response rate (PR or better) was 76.9% in the TN WM group and 83.3% in the R/R WM group, and 30.8% in TN WM group and 66.7% in the R/R WM group achieved VGPR or better. In the WM group, serum immunoglobulin M (IgM) levels decreased, and hemoglobin levels increased over time (Supplementary Fig. 2).

At the earlier data cutoff of 10 May 2022, IRC-assessed ORR (PR-L or better), the primary endpoint, was 100% in the TN, R/R, and overall CLL/SLL groups, as previously reported (Supplementary Fig. 3A) [20], with a median follow-up of 16.0 and 17.7 months in the TN and R/R CLL/SLL groups, respectively. Two patients with R/R CLL were enrolled after the data cutoff. In the WM group, IRC-assessed ORR (minor response or better; the primary endpoint) at the 10 May 2022 data cutoff was 92.3% in the TN group, 100% in the R/R group, and 94.7% in the overall WM group in Part 2, with a median follow-up of 14.8 months in the TN WM group and 15.0 months in the R/R WM group (Supplementary Fig. 3B) [21]. The ORRs in the TN and overall WM groups in Part 2 were statistically significantly different from the historical ORR of 52%, with a 1-sided p-value of 0.0026 in the TN and < 0.0001 in the overall WM group. At the data cutoff of 10 May 2022, ORR showed high concordance between IRC- and investigator-assessed data in both CLL/SLL and WM groups (Supplementary Table 1).

### Duration of response

At the 10 May 2023 data cutoff, the median investigator-assessed DoR had not yet been reached in the TN CLL/SLL group and was 23.3 months in the R/R CLL/SLL group. The 12-month event-free rates were 64.3% (95% CI, 34.3–83.3%) and 100% in the TN CLL/SLL and R/R CLL/SLL groups, respectively.

In patients with WM, the median investigator-assessed DoR had not yet been reached in either the TN or R/R groups. The 12-month event-free rate was 100% in both the TN and R/R WM groups.

### Time to response

Investigator-assessed median time to overall response (PR-L or better) was 2.8 months (range, 2.7–22.3 months) in the TN CLL/SLL group and 2.8 months (range, 2.8–16.6 months) in the R/R CLL/SLL group.

In the WM group, investigator-assessed median time to overall response (minor response or better) was 2.3 months (range, 1.0–5.6 months) in the TN group and 2.7 months (range, 0.9–2.8 months) in the R/R group.

### Progression-free survival

**Fig. 3:**
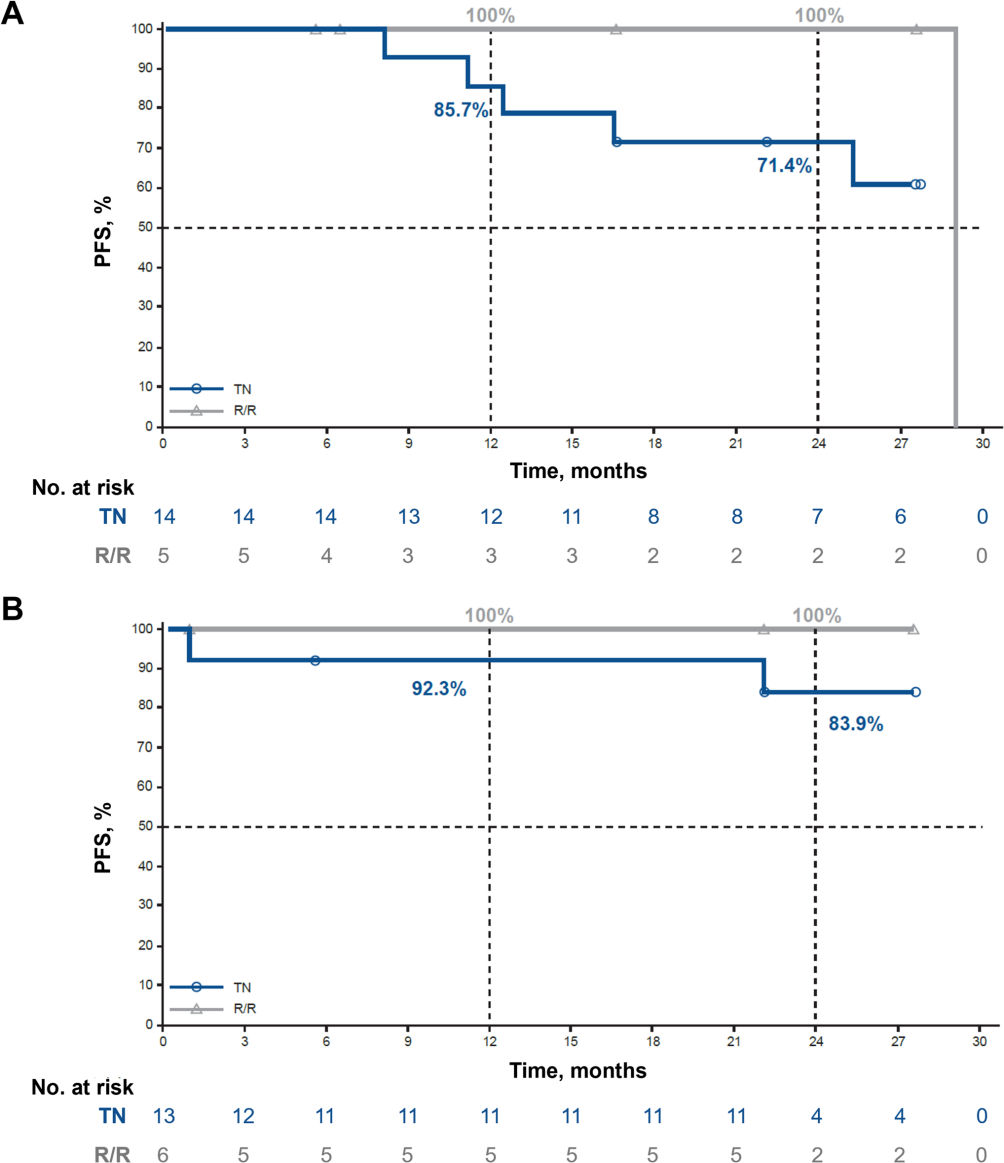
Investigator-Assessed PFS in Part 2 Patients With **A** CLL/SLL and **B** WM^a^. ^a^ Data cutoff: 10 May 2023. *CLL/SLL* chronic lymphocytic leukemia/small lymphocytic lymphoma; *PFS* progression-free survival; *R/R* relapsed/refractory; *TN* treatment naive; *WM* Waldenström macroglobulinemia

The investigator-assessed PFS event-free rate in the TN CLL/SLL group was 85.7% (95% CI, 53.9–96.2%) at 12 months and 71.4% (95% CI, 40.6–88.2%) at 24 months. In the R/R CLL/SLL group, the event-free rate was 100% at 12 and 24 months (Fig. 3A).

The investigator-assessed PFS event-free rate in the TN WM group was 92.3% (95% CI, 56.6–98.9%) at 12 months and 83.9% (95% CI, 49.4–95.7%) at 24 months. In the R/R WM group, the event-free rate was 100% at 12 and 24 months (Fig. 3B).

### Overall survival

Three patients (15.8%) in the CLL/SLL group (TN, n = 2 [14.3%]; R/R, n = 1 [20.0%]) died, all due to AEs (2 deaths within 30 days of last dose). The OS rate at 12 months was 100% in both groups; at 24 months, the OS rate was 92.9% (95% CI, 59.1–99.0%) in the TN CLL/SLL group and 100% in the R/R CLL/SLL group.

One patient (16.7%) with R/R WM died due to an AE (> 30 days after the last dose), and no deaths occurred in patients with TN WM. The OS rate at 12 and 24 months was 100% in the TN WM group and 83.3% (95% CI, 27.3–97.5%) in the R/R WM group.

### Safety

The median duration of treatment was 23.8 months (range, 0.5–32.4 months) in the overall patient population (Table 2). No DLT events were reported in the 6 patients enrolled in Part 1. At least 1 TEAE occurred in 96.4% of patients. Table 2 summarizes TEAEs and TEAEs that occurred in ≥ 10% of patients overall. The most common TEAEs observed in the overall group of patients were platelet count decreased and pyrexia (both 18.2%), COVID-19 (14.5%), and neutrophil count decreased (12.7%). The most common grade ≥ 3 TEAEs were neutrophil count decreased (10.9%), platelet count decreased (9.1%), neutropenia (5.5%), amylase increased (3.6%), cellulitis (3.6%), COVID-19 pneumonia (3.6%), and decreased appetite (3.6%) (Supplementary Table 2). TEAEs led to treatment discontinuation in 8 patients (14.5%) and death in 5 patients (9.1%). The most common TEAEs of special interest observed in the overall group were infection (54.5%), hemorrhage (45.5%), thrombocytopenia (20.0%), and neutropenia (18.2%) (Table 3). Atrial fibrillation was reported in 2 patients (3.6%); no instances were grade ≥ 3. There were 9 patients (16.4%) with COVID-19 related TEAEs. TEAEs leading to death included COVID-19 pneumonia (related to treatment, n = 1), unknown cause, drowning, septic shock, and oropharyngeal cancer (each considered unrelated to treatment by the investigator; n = 1 each), and 1 patient with R/R WM had an AE of skin angiosarcoma > 30 days after the last dose (not treatment related).

**Table 2:** Safety Summary in Patients With CLL/SLL and WM^a^

	CLL/SLL			WM			Total (N = 55)^b^
	TN (n = 14)	R/R (n = 5)	All (n = 19)	TN (n = 13)	R/R (n = 8)^c^	All (n = 21)^c^	
Duration of treatment, median (range), mo	24.1 (7.2–31.7)	18.4 (6.3–32.4)	23.0 (6.3–32.4)	25.1 (0.9–29.7)	25.4 (0.5–31.5)	25.1 (0.5–31.5)	23.8 (0.5–32.4)
≥ 1 TEAE, n (%)	14 (100)	4 (80.0)	18 (94.7)	13 (100)	7 (87.5)	20 (95.2)	53 (96.4)
Serious TEAEs, n (%)	6 (42.9)	1 (20.0)	7 (36.8)	0	4 (50.0)	4 (19.0)	18 (32.7)
TEAEs leading to discontinuation, n (%)	3 (21.4)	1 (20.0)	4 (21.1)	1 (7.7)	0	1 (4.8)	8 (14.5)
TEAEs leading to death^d^, n (%)	2 (14.3)	1 (20.0)	3 (15.8)	0	0	0	5 (9.1)
TEAEs occurring in ≥ 10% of patients overall, n (%)							
Platelet count decreased	3 (21.4)	0	3 (15.8)	0	4 (50.0)	4 (19.0)	10 (18.2)
Pyrexia	3 (21.4)	0	3 (15.8)	2 (15.4)	1 (12.5)	3 (14.3)	10 (18.2)
COVID-19	4 (28.6)	1 (20.0)	5 (26.3)	1 (7.7)	1 (12.5)	2 (9.5)	8 (14.5)
Neutrophil count decreased	2 (14.3)	1 (20.0)	3 (15.8)	0	3 (37.5)	3 (14.3)	7 (12.7)
Anemia	3 (21.4)	1 (20.0)	4 (21.1)	0	2 (25.0)	2 (9.5)	6 (10.9)
Arthralgia	0	0	0	3 (23.1)	2 (25.0)	5 (23.8)	6 (10.9)
Back pain	1 (7.1)	0	1 (5.3)	2 (15.4)	0	2 (9.5)	6 (10.9)
Constipation	3 (21.4)	1 (20.0)	4 (21.1)	0	1 (12.5)	1 (4.8)	6 (10.9)
Nasopharyngitis	1 (7.1)	1 (20.0)	2 (10.5)	3 (23.1)	1 (12.5)	4 (19.0)	6 (10.9)
Purpura	2 (14.3)	0	2 (10.5)	1 (7.7)	3 (37.5)	4 (19.0)	6 (10.9)

^a^Data cutoff: 10 May 2023

^b^Includes patients from Part 1 (FL, n = 2; MZL, n = 1; MCL, n = 1) and the Part 2 R/R MCL cohort (n = 11)

^c^Includes 2 patients with WM from Part 1

^d^Includes death (unknown cause; TN CLL/SLL, n = 1), drowning (TN CLL/SLL, n = 1), COVID-19 pneumonia (R/R CLL/SLL, n = 1), septic shock (R/R MCL, n = 1), and oropharyngeal cancer (R/R FL, n = 1)

*CLL/SLL* chronic lymphocytic leukemia/small lymphocytic lymphoma; *FL* follicular lymphoma; *MCL* mantle cell lymphoma; *MZL* marginal zone lymphoma; *R/R* relapsed/refractory; *TEAE* treatment-emergent adverse event; *TN* treatment naive; *WM* Waldenström macroglobulinemia

**Table 3:** AESIs in Patients With CLL/SLL and WM^a^

	CLL/SLL			WM			Total (N = 55)^b^
	TN (n = 14)	R/R (n = 5)	All (n = 19)	TN (n = 13)	R/R (n = 8)^c^	All (n = 21)^c^	
Any AESI, n (%)	12 (85.7)	4 (80.0)	16 (84.2)	10 (76.9)	7 (87.5)	17 (81.0)	44 (80.0)
Anemia	3 (21.4)	1 (20.0)	4 (21.1)	0	2 (25.0)	2 (9.5)	6 (10.9)
Atrial fibrillation/flutter	1 (7.1)	0	1 (5.3)	1 (7.7)	0	1 (4.8)	2 (3.6)
Hemorrhage	6 (42.9)	2 (40.0)	8 (42.1)	6 (46.2)	5 (62.5)	11 (52.4)	25 (45.5)
Major hemorrhage	3 (21.4)	1 (20.0)	4 (21.1)	0	0	0	6 (10.9)
Hypertension	0	0	0	1 (7.7)	2 (25.0)	3 (14.3)	5 (9.1)
Infection	10 (71.4)	3 (60.0)	13 (68.4)	7 (53.8)	4 (50.0)	11 (52.4)	30 (54.5)
Opportunistic infection	0	0	0	0	1 (12.5)	1 (4.8)	1 (1.8)
Neutropenia	3 (21.4)	1 (20.0)	4 (21.1)	0	4 (50.0)	4 (19.0)	10 (18.2)
Secondary primary malignancy	2 (14.3)	1 (20.0)	3 (15.8)	0	1 (12.5)	1 (4.8)	6 (10.9)
Skin cancer	1 (7.1)	0	1 (5.3)	0	1 (12.5)	1 (4.8)	2 (3.6)
Thrombocytopenia	3 (21.4)	0	3 (15.8)	0	4 (50.0)	4 (19.0)	11 (20.0)

*AESI* treatment-emergent adverse event of special interest; *CLL/SLL* chronic lymphocytic leukemia/small lymphocytic lymphoma; *FL* follicular lymphoma; *MCL* mantle cell lymphoma; *MZL* marginal zone lymphoma; *R/R* relapsed/refractory; *TN* treatment naive; *WM* Waldenström macroglobulinemia

^a^Data cutoff: 10 May 2023

^b^Includes patients from Part 1 (FL, n = 2; MZL, n = 1; MCL, n = 1) and the Part 2 R/R MCL cohort (n = 11)

^c^Includes 2 patients with WM from Part 1

## Discussion

BGB-3111-111 is the first study of the efficacy and safety of zanubrutinib in Japanese patients with TN CLL/SLL, R/R CLL/SLL, TN WM, and R/R WM. In this study, zanubrutinib was shown to be effective and tolerable in Japanese patients; the ORR was high in all cohorts (92−100%). Zanubrutinib has also been shown to be highly effective and have a sustained response in these patient populations in multiple global studies, including 3 phase 3 studies (SEQUOIA, ALPINE, and ASPEN) [9–11]. Zanubrutinib is the only BTK inhibitor to have shown superiority over ibrutinib in patients with R/R CLL/SLL in a head-to-head phase 3 study [10].

The demographic and disease characteristics of the Japanese patients with CLL/SLL enrolled in this study had several notable differences in comparison to the global populations of the ALPINE and SEQUOIA studies. The ECOG PS in Japanese patients was notably lower than that in global studies (ECOG PS of 0: 85.7% of Japanese patients with TN CLL/SLL versus 45.6% of patients in SEQUOIA [9]; 100% of Japanese patients versus 39.4% of patients with R/R CLL/SLL in ALPINE) [10]. Del(17p) was present in 13.8% of patients with R/R CLL/SLL in ALPINE [10], and SEQUOIA had separate cohorts of patients with and without del(17p) [9]; del(17p) was not present in Japanese patients with CLL/SLL in BGB-3111-111. Among Japanese patients with R/R CLL/SLL, 40% (2/5) had unmutated IGHV versus 73% (239/327) in ALPINE [10]. In patients with TN CLL/SLL, 36% (5/14) of Japanese patients had unmutated IGHV versus 53% (125/234) in SEQUOIA [9]. The lower prevalence of risk factors in Japanese patients may contribute to better outcomes compared with patients in global studies.

In the head-to-head phase 3 ALPINE trial (NCT03734016) of zanubrutinib versus ibrutinib in patients with R/R CLL/SLL, zanubrutinib demonstrated a superior ORR and PFS compared with the first-generation BTK inhibitor ibrutinib [10, 22]. In a planned interim analysis of the study (median follow-up, 15 months), zanubrutinib demonstrated superiority over ibrutinib in the primary endpoint of investigator-assessed ORR (PR or better, 78.3% versus 62.5%, respectively) [22]. Later, in the predefined final analysis of PFS (median study follow-up, 29.6 months), zanubrutinib continued to demonstrate an improved ORR (83.5% versus 74.2% in patients treated with ibrutinib) and demonstrated superiority over ibrutinib in the key secondary endpoint of PFS (hazard ratio [HR], 0.65; 95% CI, 0.49–0.86; *P* = 0.002) [10]. Patients treated with zanubrutinib had a 24-month PFS rate of 78.4%, and median PFS was not reached. Consistent results were observed with longer follow-up (median study follow-up, 42.5 months), where zanubrutinib showed PFS benefit over ibrutinib across subgroups with high-risk features [23]. Similar results are shown here in Japanese patients with R/R CLL/SLL, who had an ORR of 100% and a PFS rate of 100% at 12 and 24 months. The ORR observed at the data cutoff of 10 May 2022 was high, with a median follow-up of 17.7 months (100% in R/R CLL/SLL by investigator), and has further deepened over time.

In the phase 3 SEQUOIA trial (NCT03336333), zanubrutinib was shown to be highly effective in patients with TN CLL/SLL, regardless of del(17p) mutation status, and significantly improved PFS compared with bendamustine and rituximab (BR) (HR, 0.42; 95% CI, 0.28–0.63; *P* < 0.0001) at a median study follow-up of 26.2 months [9]. In SEQUOIA, investigator-assessed PFS at 24 months was higher in patients without del(17p) who were treated with zanubrutinib (87.7%) versus patients treated with BR (76.5%) and was similar in patients with del(17p) who were treated with zanubrutinib (87.0%). Investigator-assessed ORR (PR-L or better) was 97.5% with zanubrutinib versus 88.7% with BR in patients without del(17p) and 96.4% in patients with del(17p) who were treated with zanubrutinib [9]. In comparison, Japanese patients with TN CLL/SLL in BGB-3111-111 had an ORR of 100%, a median PFS that was not reached, and an event-free rate of 85.7% at 12 months and 71.4% at 24 months; these values were consistent with those in SEQUOIA. In patients with TN CLL/SLL, CR rates were 21% and 9% in Japanese patients and global patients enrolled in SEQUOIA, respectively [9]. In patients with R/R CLL/SLL, CR rates were 20% and 7% in Japanese patients and global patients enrolled in ALPINE, respectively [10]. While response rates were higher in Japanese patients with R/R CLL/SLL, a lower proportion of Japanese patients had high-risk characteristics compared with those in global studies.

The global phase 3 ASPEN trial (NCT03053440) was a head-to-head comparison of zanubrutinib versus ibrutinib in patients with WM with mutated *MYD88* in cohort 1. In ASPEN, zanubrutinib showed improved safety and similar efficacy (VGPR + CR rate and PFS) compared with the first-generation BTK inhibitor ibrutinib [11]. At a median study follow-up of 44.4 months, patients treated with zanubrutinib had fewer investigator-assessed PFS events than patients treated with ibrutinib (HR, 0.63; 95% CI, 0.36–1.12); the investigator-assessed PFS rate was 78.3% with zanubrutinib and 69.7% with ibrutinib at 42 months [11]. Zanubrutinib also had an investigator-assessed ORR of 95.1% versus 93.9% in patients treated with ibrutinib, with a larger difference detected in VGPR + CR (25.3% with ibrutinib and 36.3% with zanubrutinib) [11]. ORR and PFS were similar between Japanese patients with WM enrolled in BGB-3111–111 and global patients with WM enrolled in ASPEN. ORR was 92.3% in Japanese patients with TN WM, 100% in Japanese patients with R/R WM, and 94.7% in all Japanese patients with WM both at the 10 May 2022 data cutoff (median follow-up, 14.8 months) and the 10 May 2023 data cutoff (median follow-up, 26.8 months). This earlier ORR was compared against the historical control rate [19] of 52% and found to be significantly higher (*P* = 0.0026 for TN WM; *P* < 0.0001 for overall WM). Looking at the quality of response, the elevated IgM levels decreased over time and hemoglobin levels increased, suggesting an improvement of anemia. Median PFS was not reached, and the 24-month PFS rate was 83.9%, 100.0%, and 88.1% in the TN, R/R, and overall WM groups, respectively.

Zanubrutinib demonstrated consistent efficacy and tolerability and had a similar safety profile compared with those in the global studies (ALPINE [10], SEQUOIA [9], and ASPEN [11]), with no new safety signals emerging with prolonged treatment and longer follow up time. In the ALPINE study, zanubrutinib demonstrated a more favorable safety profile compared with ibrutinib in R/R CLL/SLL, with lower rates of grade ≥ 3 and serious AEs and fewer AEs leading to treatment discontinuation or dose reduction [10]. A statistically lower incidence of atrial fibrillation was demonstrated through predefined analysis (2.5% with zanubrutinib and 10.1% with ibrutinib; 2-sided *P* = 0.001); the rate of major hemorrhage was low in the zanubrutinib arm (2.9% versus 3.9% in those treated with ibrutinib) [22]. Zanubrutinib also demonstrated better tolerability compared with BR in the SEQUOIA study in patients with TN CLL/SLL, including lower rates of grade ≥ 3 and serious AEs in long-term follow-up [9]. In patients with symptomatic WM in the ASPEN trial, zanubrutinib demonstrated a more favorable safety profile compared with ibrutinib; rates of AEs, including cardiovascular AEs, were lower with zanubrutinib [11].

Overall, the rates of TEAEs leading to treatment discontinuation or death were comparable in global and Japanese patients with CLL/SLL and WM. Given the smaller sample size for the Japanese study and the shorter follow-up time, differences in AE incidence may be observed when compared with the larger randomized trials. In Japanese patients with TN CLL/SLL: 3 of 14 patients (21.4%) discontinued treatment due to AEs, while 20 patients (8.3%) discontinued treatment due to AEs in SEQUOIA [9]. Among Japanese patients with R/R CLL/SLL, 1 of 5 patients (20%) discontinued treatment due to AEs, and 50 (15.4%) discontinued due to AEs in ALPINE [10]. Among Japanese patients with WM, 1 of 21 patients discontinued treatment due to AEs (4.8%), while 9 (8.9%) discontinued treatment due to AEs in ASPEN [11]. Atrial fibrillation/flutter in the BGB-3111–111 study occurred in 1 patient (5.3%) with CLL/SLL and 1 patient (4.8%) with WM; the incidence was similar in the global ALPINE (5.2%) [10], SEQUOIA (3.3%) [9], and ASPEN (7.9%) studies [11]. Overall, the incidences of secondary primary malignancies were similar among Japanese patients (10.9%) and the patients in the global studies (ALPINE 12.3% [10], SEQUOIA 15.7% [9], ASPEN 16.8% [11]). In the R/R CLL/SLL group, secondary primary malignancy was reported in 1 of 5 patients (gastric cancer: the patient’s gastrointestinal endoscopy identified a mucosal abnormality requiring follow-up prior to the first dose) representing 20% versus 12.3% of patients with R/R CLL/SLL in the ALPINE study, however, the difference in incidence may be due to the small sample size [10]. Nevertheless, monitoring for signs of other cancers is warranted. Major hemorrhage occurred in 4 of 19 patients (21.1%) with CLL/SLL in the BGB-3111-111 study versus 3.7% in ALPINE [10] and 5.0% in SEQUOIA [9]. Of patients with CLL/SLL who experienced major hemorrhage, 2 were receiving antithrombotic therapy (rivaroxaban and aspirin), 2 had causes of hemorrhage (trauma and endoscopic submucosal dissection), 1 had hyphemia after multiple laser photocoagulation for diabetic retinopathy, all were not considered related to study treatment, and none of the 4 events led to treatment discontinuation. In the overall population, 6 of 55 patients (10.9%) experienced major hemorrhage and 1 case led to treatment discontinuation; Given the known risk for bleeding in patients treated with BTK inhibitors, physicians should account for concomitant medications and procedures that may increase the risk of hemorrhage when treating this population. Overall, the safety profile of zanubrutinib in Japanese patients did not significantly differ from the profile seen in the global studies, and the overall results suggest that zanubrutinib is well tolerated and has a safety profile in the Japanese population consistent with that in the rest of the world.

This study had several limitations. The overall low number of enrolled patients compared with global studies may limit the applicability of the findings to the broader population of patients in Japan with TN CLL/SLL, R/R CLL/SLL, or WM. The BGB-3111-111 study was conducted during the start of the COVID-19 pandemic (the first patient received zanubrutinib on 30 January 2020, and the data cutoff was 10 May 2023), and the safety profile may have been impacted by patients being vaccinated for COVID-19 only later in the study. COVID-19 infections were reported in 8 of 55 patients (14.5%) during this period. Longer follow-up is needed to evaluate the findings for DoR, PFS, and OS.

## Conclusions

In this analysis, with a median follow-up of more than 2 years, zanubrutinib demonstrated high response rates and durable disease response in Japanese patients with CLL/SLL or WM. Zanubrutinib was generally well tolerated, with no new safety signals observed. Hemorrhage is a known risk of BTK inhibitors, especially when administered with concomitant antiplatelet or anticoagulant therapies, therefore such concomitant medications and medical procedures should be provided with caution of the risk of hemorrhage in this population. Safety and efficacy data are comparable to those observed in published data from global studies. The results from this phase 1/2 study suggest that zanubrutinib can be a valuable treatment option for Japanese patients with CLL/SLL or WM.

## Supplementary Information

Below is the link to the electronic supplementary material.

Supplementary file1 (DOCX 415 KB)
