# Supplementary material for: Zanubrutinib in Japanese treatment-naive and relapsed/refractory patients with Waldenström macroglobulinemia and CLL/SLL
Source: Int J Hematol. 2025 Feb 17;121(4):483–93. doi: 10.1007/s12185-025-03925-1 (PMC11922998; doi:10.1007/s12185-025-03925-1)
Supplement: Supplementary file 1 — Supplementary file1 (DOCX 415 KB) [file 12185_2025_3925_MOESM1_ESM.docx]

Supplementary Materials – Table of Contents

**Supplementary Figure 1.** Study Design

**Supplementary Methods:** Selection of Study Population

**Supplementary Methods:** Statistical Analysis

**Supplementary Methods:** Adverse Events

**Supplementary Methods:** Dose-Limiting Toxicity

**Supplementary Figure 2**. Median IgM and Median Hemoglobin Over Time in Patients With WM in Part 2

**Supplementary Figure 3**. Best Overall Response Assessed by IRC in Part 2 in Patients With (A) CLL/SLL and (B) WM

**Supplementary Table 1.** Concordance Data for Investigator and IRC Across CLL/SLL and WM Subgroups

**Supplementary Table 2.** Grade ≥3 TEAEs in Patients With CLL/SLL and WM


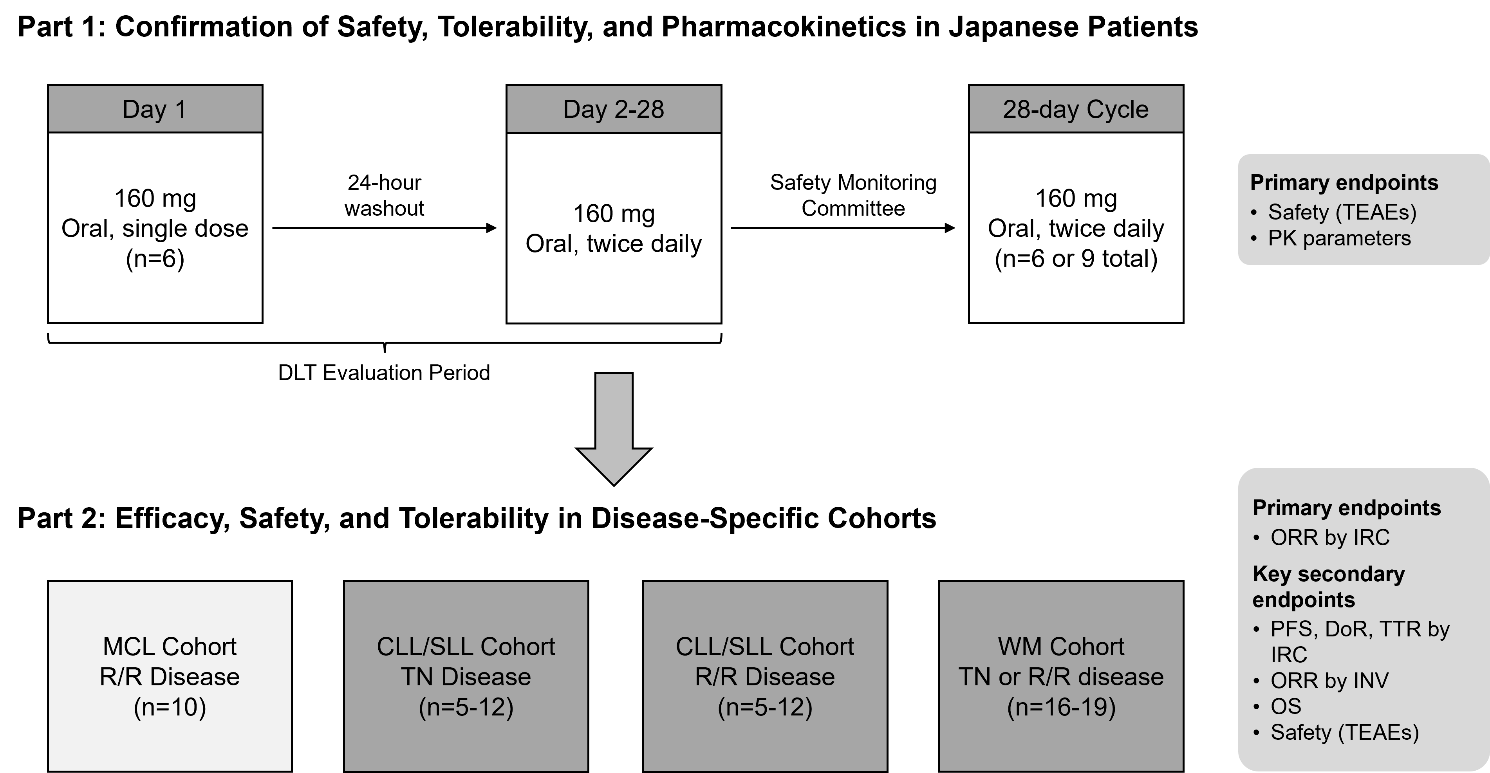
Supplementary Figure 1. Study Design

Data cutoff: 10 May 2023.

CLL/SLL, chronic lymphocytic leukemia/small lymphocytic lymphoma; DLT, dose-limiting toxicity; DoR, duration of response; INV, investigator; IRC, independent review committee; MCL, mantle cell lymphoma; ORR, overall response rate; OS, overall survival; PFS, progression-free survival; PK, pharmacokinetics; R/R, relapsed/refractory; TEAE, treatment-emergent adverse event; TN, treatment naive; TTR, time to response; WM, Waldenström macroglobulinemia.

Supplementary Methods: Selection of Study Population

***Inclusion criteria***

Patients eligible to participate in this study must meet all the following criteria:

1. Written informed consent, signed and dated prior to any study-specific procedures
2. Japanese and aged ≥20 years at time of study entry
3. In Part 1, no standard treatment option in the judgment of the physician, in addition to meeting the following diagnostic criteria
4. Confirmed diagnosis of only 1 of the following:
   1. Chronic lymphocytic leukemia/small lymphocytic lymphoma (CLL/SLL) diagnosis that meets the International Workshop on Chronic Lymphocytic Leukemia (iwCLL) criteria (Hallek M, et al. *Blood*. 2018;131(25):2745-60)
      1. Part 1: relapsed/refractory (R/R) disease, defined as disease that relapsed after or was refractory to at least 1 prior therapy for CLL/SLL
      2. Part 2: treatment-naive (TN) or R/R disease, defined as disease that relapsed after or was refractory to at least 1 prior therapy for CLL/SLL
      3. Requiring treatment as defined by at least 1 of the following criteria:
         1. Evidence of progressive marrow failure as manifested by the development or worsening of anemia and/or thrombocytopenia
         2. Massive (ie, ≥6.0 cm below left costal margin), progressive, or symptomatic splenomegaly
         3. Massive nodes (ie, ≥10.0 cm in greatest diameter) or progressive or symptomatic lymphadenopathy
         4. Progressive lymphocytosis with an increase of ≥50% over a 2-month period or lymphocyte doubling time of <6 months. Lymphocyte doubling time may be determined by linear regression extrapolation of absolute lymphocyte counts obtained at intervals of 2 weeks over an observation period of 2 to 3 months. In patients with initial blood lymphocyte counts of <30×10^9^/L (30,000/μL), lymphocyte doubling time should not be used as a single parameter to define treatment indication. In addition, factors contributing to lymphocytosis or lymphadenopathy other than CLL/SLL (eg, infection, steroid administration) should be excluded
         5. Symptomatic or functional extranodal involvement (eg, skin, kidney, lung, spine)
         6. Constitutional symptoms, defined as 1 or more of the following disease-related symptoms or signs:
            1. Unintentional loss of ≥10% of body weight within the previous 6 months
            2. Significant fatigue (ie, inability to work or perform usual activities)
            3. Fever of ≥100.5°F or ≥38°C for ≥ 2 weeks without other evidence of infection
            4. Night sweats for ≥1 month without evidence of infection
   2. Mantle cell lymphoma
      1. Parts 1 and 2: previously received 1 or more lines of therapy, with documented failure to achieve at least partial response or with documented disease progression after the most recent systemic treatment
      2. Measurable disease by computed tomography (CT)/magnetic resonance imaging (MRI), defined as at least 1 lymph node measuring >1.5 cm in greatest diameter and measurable in 2 perpendicular dimensions
   3. Waldenström macroglobulinemia
      1. Part 1: R/R disease, defined as disease that relapsed after or was refractory to at least 1 prior therapy for Waldenström macroglobulinemia (WM)
      2. Part 2: TN or R/R disease, defined as disease that relapsed after or was refractory to at least 1 prior therapy for WM
      3. Measurable disease, defined as serum immunoglobulin M level of >0.5 g/dL
      4. At least 1 of the following criteria for requiring WM treatment (Dimopoulos MA, et al. *Blood.* 2014;124(9):1404-11):
         1. Recurrent fever, night sweats, weight loss, fatigue
         2. Hyperviscosity
         3. Lymphadenopathy that is either symptomatic or bulky (≥5 cm in maximum diameter)
         4. Symptomatic hepatomegaly and/or splenomegaly
         5. Symptomatic organomegaly and/or organ or tissue infiltration
         6. Peripheral neuropathy due to WM
         7. Symptomatic cryoglobulinemia
         8. Cold agglutin anemia
         9. Immune hemolytic anemia and/or thrombocytopenia
         10. Nephropathy related to WM
         11. Amyloidosis related to WM
         12. Hemoglobin of ≤10 g/dL
         13. Platelet count of <100×10^9^/L
   4. Marginal zone lymphoma (eligible for Part 1 only)
      1. R/R splenic and/or nodal disease, defined as disease that relapsed after or was refractory to at least 1 prior therapy for splenic and/or nodal marginal zone lymphoma
      2. Active disease requiring treatment
      3. Measurable disease by CT/MRI, defined as at least 1 lymph node measuring >1.5 cm in greatest diameter and measurable in 2 perpendicular dimensions
   5. Follicular lymphoma (eligible for Part 1 only)
      1. R/R follicular lymphoma (grade 1, 2, or 3a based on the World Health Organization 2008 classification of tumors of hematopoietic and lymphoid tissue), defined as disease that relapsed after or was refractory to at least 1 prior systemic therapy for follicular lymphoma
      2. Measurable disease by CT/MRI, defined as at least 1 lymph node measuring >1.5 cm in greatest diameter and measurable in 2 perpendicular dimensions
5. Eastern Cooperative Oncology Group performance status of 0, 1, or 2
6. Adequate organ function, defined as:
   1. Absolute neutrophil count (ANC) of ≥1000 /mm^3^, except for patients with bone marrow involvement by B-cell malignancy, in whom the ANC must be ≥750/ mm^3^
   2. Platelets of ≥75×10^9^/L, except for patients with bone marrow involvement by B-cell malignancy, in whom the platelet count must be ≥50×10^9^/L
   3. Hemoglobin of >80 g/L (may be post transfusion)
   4. Creatinine clearance of ≥30 mL/min as estimated by 1 of the following:
      1. Cockcroft-Gault equation: (140 – age) × mass (kg)/72 × creatinine (mg/dL); multiply by 0.85 in female individuals
      2. CKD-EPI equation
      3. Nuclear medicine scan
      4. 24-hour urine collection
   5. Aspartate aminotransferase/serum glutamic-oxaloacetic transaminase and/or alanine aminotransferase/serum glutamic-pyruvic transaminase level of ≤3 × upper limit of normal (ULN)
   6. Total bilirubin level of ≤2 × ULN (unless documented Gilbert syndrome)
   7. Serum amylase of ≤1.5 × ULN and/or serum lipase of ≤1.5 × ULN
7. Patients with relapse after autologous stem cell transplant are eligible when at least 3 months after transplant
8. Female patients of childbearing potential must practice highly effective methods of contraception (Protocol Section 5.3), initiated prior to first dose of study drug, for the duration of the study and for ≥90 days after the last dose of zanubrutinib
9. Male patients are eligible if they are abstinent, have undergone vasectomy, or agree to the use of barrier contraception with other methods described in Protocol Section 5.3 during the study treatment period and for ≥90 days after the last dose of zanubrutinib. Male patients must not donate sperm from initial study drug administration until 90 days after drug discontinuation
10. Life expectancy of >4 months
11. Ability to comply with the requirements of the study

***Exclusion criteria***

Patients eligible to participate in this study must ***not*** meet any of the following exclusion criteria:

1. Prior malignancy within the past 3 years, except for curatively treated basal or squamous cell skin cancer, superficial bladder cancer, carcinoma in situ of the cervix or breast, or localized Gleason score 6 prostate
2. Underlying medical conditions that, in the investigator’s opinion, will render the administration of study drug hazardous or obscure the interpretation of safety or efficacy results
3. Known central nervous system involvement by lymphoma/leukemia
4. Known plasma cell neoplasm, prolymphocytic leukemia, or history of or currently suspected Richter syndrome
5. Prior allogeneic stem cell transplant
6. Receipt of any biologic- and/or immunologic-based therapy, including experimental therapy for leukemia, lymphoma, or myeloma (including, but not limited to, monoclonal antibody therapy [eg, rituximab] and/or cancer vaccine therapy) within 4 weeks prior to first dose of zanubrutinib
7. Corticosteroid given with antineoplastic intent within 7 days prior to first dose of zanubrutinib
8. Systemic chemotherapy or radiation therapy within 2 weeks prior to first dose of zanubrutinib
9. Active fungal, bacterial, and/or viral infection requiring systemic therapy
10. Prior therapy with B-cell receptor inhibitor (eg, Bruton tyrosine kinase, phosphoinositide 3-kinase delta, and/or spleen tyrosine kinase inhibitor) or B-cell lymphoma 2 inhibitor (eg, venetoclax/ABT-199)
11. Major surgery within 4 weeks of study treatment
12. Toxicity from prior anticancer therapy that has not recovered to grade ≤1 (except for alopecia, ANC, hemoglobin, and platelet count; for ANC, hemoglobin, and platelet count, see inclusion criterion 5)
13. Clinically significant cardiovascular disease including the following:
    1. Myocardial infarction within 6 months before screening
    2. Unstable angina within 3 months before screening
    3. New York Heart Association class III or IV congestive heart failure
    4. History of clinically significant arrhythmias (eg, sustained ventricular tachycardia, ventricular fibrillation, torsades de pointes)
    5. QTcF of >480 milliseconds based on the Fredericia formula
    6. History of Mobitz II second-degree or third-degree heart block without a permanent pacemaker in place
    7. Uncontrolled hypertension as indicated by a minimum of 2 consecutive blood pressure measurements showing systolic blood pressure of >170 mm Hg and diastolic blood pressure of >105 mm Hg at screening
14. Known infection with HIV or serologic status reflecting active viral hepatitis B (HBV) or viral hepatitis C (HCV) infection:
    1. HBsAg positive, or
    2. HBcAb positive and HBV DNA detected, or
    3. Unvaccinated HBsAb positive and HBV DNA detected. Patients who are HBcAb positive or unvaccinated HBsAb positive are eligible if HBV DNA is undetectable and they are willing to undergo monthly monitoring for HBV reactivation
    4. Presence of HCV antibody. Patients with presence of HCV antibody are eligible if HCV RNA is undetectable
15. Pregnant, lactating, or nursing women; female patients who suspend nursing are allowed. Patients who wish to resume nursing after the completion of treatment should wait for ≥2 weeks after the last dose of study drug (substantially >5 half-lives of zanubrutinib)
16. Inability to swallow capsules or disease that significantly affects gastrointestinal function, such as malabsorption syndrome, resection of the stomach or small bowel, bariatric surgery procedures, symptomatic inflammatory bowel disease, or partial or complete bowel obstruction
17. Inability to comply with study procedures
18. Need for ongoing treatment with a strong CYP3A inhibitor or inducer
19. History of intracranial hemorrhage or stroke within 6 months prior to first dose of zanubrutinib
20. Severe or debilitating pulmonary disease, including active interstitial pneumonia or interstitial lung disease
21. Concurrent participation in another therapeutic clinical trial
22. Autoimmune anemia and/or thrombocytopenia that is poorly responsive to corticosteroids or other standard therapy
23. Ongoing drug-induced liver injury, alcoholic liver disease, nonalcoholic steatohepatitis, primary biliary cirrhosis, ongoing extrahepatic obstruction caused by cholelithiasis, cirrhosis of the liver, or portal hypertension
24. History of severe bleeding disorder such as hemophilia A, hemophilia B, or von Willebrand disease or history of spontaneous bleeding requiring blood transfusion or other medical intervention
25. Hypersensitivity to zanubrutinib or any of the other ingredients of the study drug

Supplementary Methods: Hypothesis Testing in Part 2 WM Cohort

The primary endpoint of overall response rate (ORR) will be analyzed in Part 2 patients with TN WM and the overall WM population in the exposed analysis set.

The ORR in the zanubrutinib and historical control groups (52% [Gertz MA, et al. *Leuk Lymphoma.* 2004;45(10):2047-55]) will be compared using a hierarchical fixed-sequence procedure under the null and alternative hypotheses:

H0: ORR=52%

H1: ORR >52%

- Testing for these hypotheses will be performed in patients with TN WM first, at a 1-sided alpha of 0.05. A binomial exact test will be performed for hypothesis testing
- If the result in the TN WM patient population is statistically significant (ie, the obtained 1-sided *P*-value is ≤.05), the primary objective will have been met and further testing will be performed in the overall WM population at a 1-sided alpha of .05.

Supplementary Methods: Adverse Events

Adverse events (AEs) were graded by the investigators using National Cancer Institute Common Terminology Criteria for Adverse Events version 5.0 (hematologic toxicity in patients with CLL/SLL was graded according to the iwCLL 2018 grading scale as shown in the table). The period for dose-limiting toxicity assessment in Part 1 was 28 days from the first dose of zanubrutinib.

| Grade^a^ | Decrease in platelets^b^ or Hgb^c^ (nadir) from pretreatment value | Absolute neutrophil count/μL^d^ (nadir) |
| --- | --- | --- |
| 0 | No change to 10% | ≥2000 |
| 1 | 11%-24% | ≥1500 and <2000 |
| 2 | 25%-49% | ≥1000 and <1500 |
| 3 | 50%-74% | ≥500 and <1000 |
| 4 | ≥75% | <500 |

Source: Hallek M, et al. *Blood* 2018.
ANC, absolute neutrophil count; CLL, chronic lymphocytic leukemia, Hgb: hemoglobin; WBC, white blood cell.

^a^ Grades: 1, mild; 2, moderate; 3, severe; 4, life-threatening; 5, fatal. Death occurring as a result of toxicity at any level of decrease from pretreatment will be reported as grade 5.

^b^ Platelet counts must be below normal levels for grades 1 to 4. If, at any level of decrease, the platelet count is <20×10^9^/L, this will be considered grade 4 toxicity, unless a severe or life-threatening decrease in the initial platelet count (eg, <20×10^9^/L) was present pretreatment, in which case the patient is not evaluable for toxicity referable to platelet counts.

^c^ Hgb levels must be below normal levels for grades 1 to 4. Baseline and subsequent Hgb determinations must be performed before any transfusions. The use of erythropoietin is irrelevant for the grading of toxicity but should be documented.

^d^ ANC of <1×10^9^/L should be judged to be grade 3 toxicity. Other decreases in the WBC or circulating granulocytes are not to be considered because a decrease in WBC is a desired therapeutic endpoint. A gradual decrease in granulocytes is not a reliable index in CLL for stepwise grading of toxicity. Patients with ANC of <1×10^9^/L before therapy are not evaluable for toxicity referable to the ANC. The use of growth factors such as granulocyte colony–stimulating factor is not relevant to the grading of toxicity but should be documented.

**Supplementary Methods: Dose-Limiting Toxicity**

In Part 1, patients were evaluated for dose-limiting toxicities (DLTs) during cycle 1. During cycle 1 and on a case-by-case basis and with sponsor approval, patients were hospitalized based on investigator decision. A DLT was defined as a toxicity or AE meeting at least 1 of the following criteria and that was not due to alternative causes (eg, disease progression, underlying illness, concurrent illness, or concomitant medication): grade 4 neutropenia for >10 days or grade ≥3 neutropenia with fever or infection (the use of growth factor support to avoid a DLT of neutropenia was not permitted); grade 4 thrombocytopenia for >10 days or grade ≥3 thrombocytopenia with clinically significant bleeding (grade ≥3 thrombocytopenia requiring transfusion was considered a DLT); grade ≥3 atrial fibrillation associated with hemodynamic instability; grade ≥3 hemorrhage; grade ≥3 opportunistic infection; or any grade ≥2 nonhematologic toxicity resulting in ≥14 days of study drug interruption in a cycle or permanent treatment discontinuation.

After the first 6 patients were treated for 28 days, enrollment was paused while a safety monitoring committee composed of participating investigators and the sponsor’s internal medical and safety personnel reviewed the safety and tolerability data from these 6 patients.

Zanubrutinib was considered safe and tolerable if the incidence of DLTs was <33% (ie, ≤1 of 6 patients experience a DLT). Zanubrutinib would be considered intolerable if ≥50% (ie, ≥3 of 6 patients) experienced a DLT. If DLTs occured in ≥33% but <50% of the first 6 patients enrolled in Part 1 (ie, 2 of 6 patients experienced a DLT), 3 additional patients would be enrolled. If DLTs occured in ≤2 of the 9 patients enrolled in Part 1, zanubrutinib would be considered safe and tolerable. If DLTs occured in 3 of the 9 patients enrolled in Part 1, the study safety monitoring committee would evaluate the cases and decide whether the study could continue. If ≥4 of 9 patients experienced a DLT, zanubrutinib would be considered intolerable.

Supplementary Figure 2. Median IgM and Median Hemoglobin Over Time in Patients With WM in Part 2


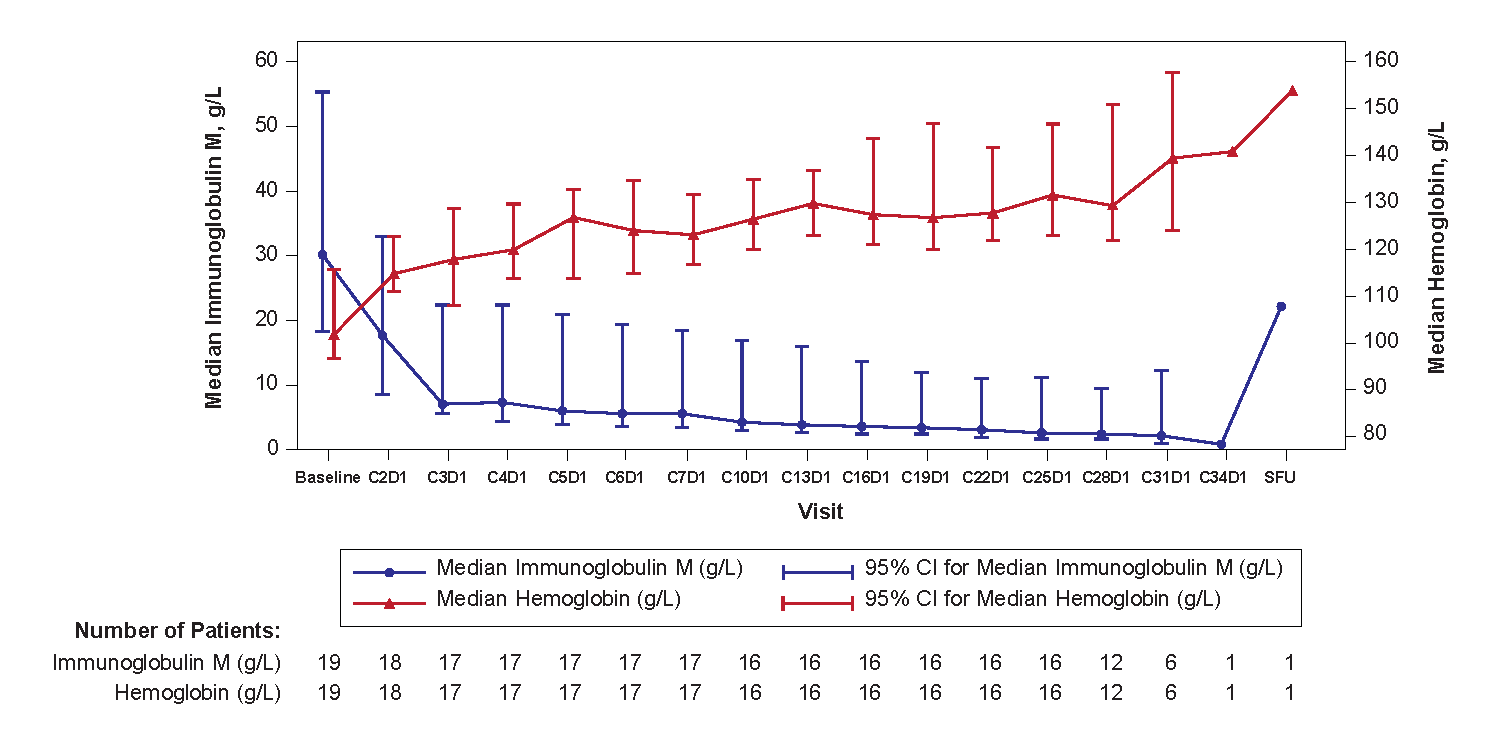


C, cycle; D, day; IgM, immunoglobulin M; SFU, safety follow-up; WM, Waldenström macroglobulinemia.

Supplementary Figure 3. Best Overall Response Assessed by IRC in Part 2 Patients With (A) CLL/SLL and (B) WM^a^
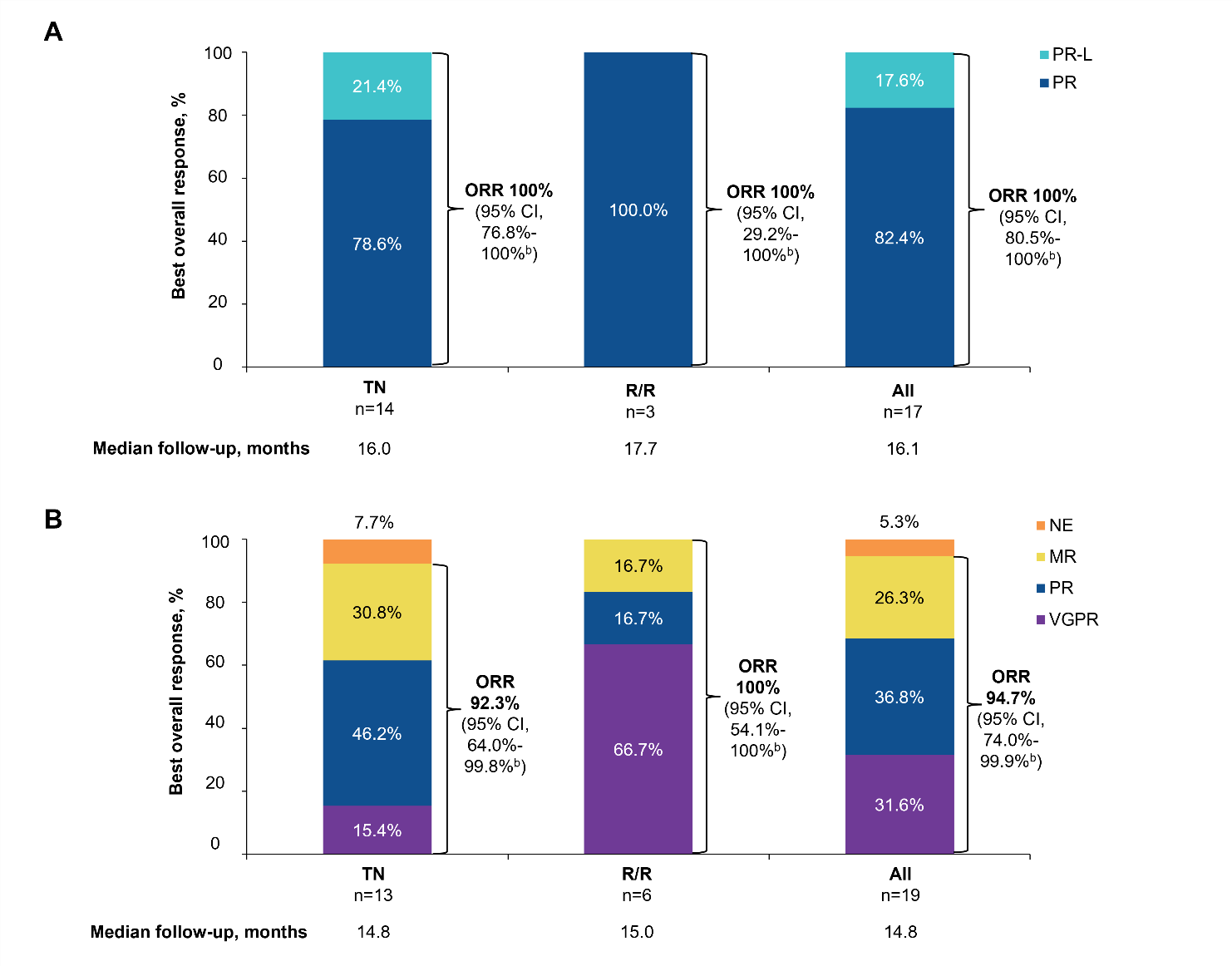


^a^ Data cutoff: 10 May 2022.

^b^ Estimated using Clopper-Pearson method.

CLL/SLL, chronic lymphocytic leukemia/small lymphocytic lymphoma; MR, minor response; NE, not evaluable; ORR, overall response rate; PR, partial response; PR-L, partial response with lymphocytosis; R/R, relapsed/refractory; TN, treatment naive; VGPR, very good partial response; WM, Waldenström macroglobulinemia.

Supplementary Table 1. Concordance Data for Investigator and Independent Review Committee Across CLL/SLL and WM Subgroups^a^

|  | **CLL/SLL** | | | **WM** | | |
| --- | --- | --- | --- | --- | --- | --- |
|  | **TN**  **(n=14)** | **R/R**  **(n=3)** | **All**  **(N=17)** | **TN**  **(n=13)** | **R/R**  **(n=6)** | **All**  **(N=19)** |
| **Concordance, %** | 92.9 | 100.0 | 94.1 | 100.0 | 100.0 | 100.0 |
| Overall response by investigator, n | 13 | 3 | 16 | 12 | 6 | 18 |
| Overall response by independent review committee, n | 14 | 3 | 17 | 12 | 6 | 18 |

^a^ Data cutoff: 10 May 2022.

CLL/SLL, chronic lymphocytic leukemia/small lymphocytic lymphoma; R/R, relapsed/refractory; TN, treatment naive; WM, Waldenström macroglobulinemia.

Supplementary Table 2. Grade ≥3 TEAEs in Patients With CLL/SLL and WM^a^

|  | **CLL/SLL** | | | **WM** | | | **Total**  **(N=55)^b^** |
| --- | --- | --- | --- | --- | --- | --- | --- |
| **n (%)** | **TN**  **(n=14)** | **R/R**  **(n=5)** | **All**  **(n=19)** | **TN**  **(n=13)** | **R/R**  **(n=8)** | **All**  **(n=21)^c^** |  |
| **Grade ≥3 TEAEs** | 7 (50.0) | 1 (20.0) | 8 (42.1) | 2 (15.4) | 6 (75.0) | 8 (38.1) | 26 (47.3) |
| **Grade ≥3 TEAEs occurring in** ≥**3% of patients overall** | | | | | | |  |
| Neutrophil count decreased | 2 (14.3) | 1 (20.0) | 3 (15.8) | 0 | 3 (37.5) | 3 (14.3) | 6 (10.9) |
| Platelet count decreased | 3 (21.4) | 0 | 3 (15.8) | 0 | 2 (25.0) | 2 (9.5) | 5 (9.1) |
| Neutropenia | 1 (7.1) | 0 | 1 (5.3) | 0 | 1 (12.5) | 1 (4.8) | 3 (5.5) |
| Amylase increased | 0 | 0 | 0 | 1 (7.7) | 0 | 1 (4.8) | 2 (3.6) |
| Cellulitis | 1 (7.1) | 0 | 1 (5.3) | 0 | 0 | 0 | 2 (3.6) |
| COVID-19 pneumonia | 0 | 1 (20.0) | 1 (5.3) | 0 | 0 | 0 | 2 (3.6) |
| Decreased appetite | 1 (7.1) | 0 | 1 (5.3) | 0 | 1 (12.5) | 1 (4.8) | 2 (3.6) |

^a^ Data cutoff: 10 May 2023.

^b^ Includes patients from Part 1 (FL, n=2; MZL, n=1; MCL, n=1) and Part 2 R/R MCL cohort (n=11).

^c^ Includes 2 patients with WM from Part 1.

CLL/SLL, chronic lymphocytic leukemia/small lymphocytic lymphoma; MCL, mantle cell lymphoma; R/R, relapsed or refractory; TEAE, treatment-emergent adverse event; TN, treatment naive; WM, Waldenström macroglobulinemia.
